# Corilagin Interferes With Toll-Like Receptor 3-Mediated Immune Response in Herpes Simplex Encephalitis

**DOI:** 10.3389/fnmol.2019.00083

**Published:** 2019-04-26

**Authors:** Lu-Jun Li, Shao-Jun Zhang, Pan Liu, You-Qin Wang, Zhi-Lin Chen, Yu-Jie Wang, Jia-Bin Zhou, Yuan-Jin Guo, Lei Zhao

**Affiliations:** ^1^National & Local Joint Engineering Research Center of High-throughput Drug Screening Technology, State Key Laboratory of Biocatalysis and Enzyme Engineering, Hubei University, Wuhan, China; ^2^School of Clinical Medicine, Hubei University of Chinese Medicine, Wuhan, China; ^3^Renmin Hospital of Hubei University of Medicine, The Postgraduate Training Center of Jinzhou Medical University, Shiyan, China; ^4^Department of Infectious Diseases, Union Hospital, Tongji Medical College, Huazhong University of Science and Technology, Wuhan, China; ^5^Department of Vascular Surgery, Union Hospital, Tongji Medical College, Huazhong University of Science and Technology, Wuhan, China; ^6^Department of Neurosurgery, Affiliated Hospital of Jining Medical University and Shangdong Provincial Key Laboratory of Stem Cells and Neuro-Oncology, Jining, China; ^7^Department of Neurology, Union Hospital, Tongji Medical College, Huazhong University of Science and Technology, Wuhan, China

**Keywords:** corilagin, herpes simplex virus type 1, inflammation, signaling pathway, toll-like receptor 3

## Abstract

Herpes simplex encephalitis (HSE) is the most common infectious disease of the central nervous system worldwide. However, the pathogenesis of HSE is not clear. Research has shown that the immune response mediated by the toll-like receptor 3 (TLR3) signaling pathway is essential to protect the central nervous system against herpes simplex virus (HSV) infection. However, an excessive immune response may cause tissue damage accompanied by pathological changes. The aim of this study was to explore the molecular mechanism *via* which corilagin controls HSE through the TLR3 signaling pathway *in vitro* and *in vivo*. Cells and mice were pre-treated with polyriboinosinic polyribocytidylic acid [poly(I:C)] or HSV type 1, and then treated with corilagin. After treatment, the mRNA and protein levels of TLR3, TLR-like receptor-associated interferon factor (TRIF), tumor necrosis factor (TNF) receptor type 1-associated DEATH domain protein (TRADD), TNF receptor-associated factor (TRAF) 3 and 6, nuclear factor-kappa-B (NF-κB) essential modulator (NEMO), P38, and interferon regulatory factor 3 (IRF3) were decreased. Interleukin-6 (IL-6), TNF-α, and type 1 interferon-β were also decreased. When TLR3 expression was silenced or increased, corilagin still inhibited the expression of TLR3 and its downstream mediators. Hematoxylin-eosin (HE) staining and immunohistochemical examinations of mouse brain tissues revealed that corilagin lessened the degree of brain inflammation. Altogether, these results suggest that corilagin may regulate the immune response in HSE and relieve inflammatory injury by interfering with the TLR3 signaling pathway.

## Introduction

The herpes simplex virus (HSV) is a common human pathogen. There are two serotypes of HSV: HSV-1 and HSV-2. HSV-1 mainly invades the upper part of the body, causing infections of the brain, eyes, lips, oral cavity, and waist; HSV-2 is one of the main causes of sexually transmitted diseases. Herpes simplex encephalitis (HSE) is the most common cause of sporadic necrotizing encephalitis (Sili et al., 2014), which is mostly triggered by HSV-1 infection (James et al., 2009). The incidence of HSE is approximately one in 250,000–500,000 people per year. However, if left untreated, HSE can have a mortality rate as high as 70% (Piret and Boivin, 2015). The main treatments for HSE are antiviral drugs, adrenocortical hormones, and symptomatic and supportive care. Currently, acyclovir (ACV) is the main antiviral drug for HSE; ACV interferes with the HSV-1 DNA polymerase, thus inhibiting viral replication (Solomon et al., 2007). However, over the years, HSV-1 has developed resistance to ACV (Schulte et al., 2010; Piret and Boivin, 2011), and side effects may arise from increasing doses of antiviral drugs. Corticosteroids also play an important role in improving the symptoms of HSE (Ramos-Estebanez et al., 2014). However, the prolonged use of corticosteroids is associated with side effects (Ling et al., 1981) and is controversial (Openshaw and Cantin, 2005). The curative effect of medication is limited, even when treatment is adequate, with 45%–60% of survivors continuing to live with severe neurological dysfunction (Gnann et al., 2015). Therefore, it is important to find safer and more effective drugs that significantly improve the symptoms of HSE.

The pathogenesis of HSE is not well understood. Toll-like receptors (TLRs), as pathogen recognition receptors (PRRs), can recognize pathogen-associated molecular patterns (PAMPs), including virus particles, proteins, and nucleic acids, which can initiate the body innate immune response (Carty and Bowie, 2011; Li et al., 2018). Studies have shown that TLR2 and TLR9 play a major role in the pathogenesis of HSV-induced encephalitis (Guo et al., 2015a, b). Furthermore, TLR2-, TLR3-, and TLR9-mediated pathways contribute to the host’s antiviral immune response and tissue damage in HSE (Piret and Boivin, 2015). Previous experiments have demonstrated that the release of large amounts of pro-inflammatory cytokines during HSV infection is the main cause of brain damage in HSE (Conrady et al., 2010). TLR2 is located on the cell surface and identifies the HSV-1 that enters the body. TLR3 and TLR9 are located in endosomes; TLR3 recognizes the viral double-stranded RNA (dsRNA), while TLR9 recognizes the viral DNA (Kawai and Akira, 2010).

Corilagin is a member of the tannin family and one of the active ingredients of many herbal medicines. The chemical name of corilagin is beta-1-oxy-galloyl-3, 6(R)-hexaloxydiphenyl-D-glucose, and its molecular formula is C_27_H_22_O_18_ (Gaudreault et al., 2002; Li et al., 2018; Yang et al., 2018). Several studies have shown that corilagin exerts a variety of pharmacological effects, including antihyperalgesic (Moreira et al., 2013), antihypertensive (Cheng et al., 1995), anti-atherosclerotic (Duan et al., 2005), antitumorigenic (Hau et al., 2010), antifibrogenic (Du et al., 2016; Li et al., 2016, 2017), antioxidant and hepatoprotective (Kinoshita et al., 2007; Liu et al., 2017), and thrombolytic (Shen et al., 2003) effects. Other studies have also shown that corilagin inhibits nuclear factor kappa B (NF-κB) signaling and the production of pro-inflammatory cytokines (Zhao et al., 2008; Gambari et al., 2012; Li et al., 2017). We previously showed that corilagin inhibits nuclear transcription of NF-κB in lipopolysaccharide-stimulated mouse macrophages, and significantly reduces the release of pro-inflammatory cytokines [tumor necrosis factor (TNF)-α, interleukin (IL)-1β] and nitric oxide (NO) from HSV-stimulated BV-2 microglia (Guo et al., 2010).

The HSV is a dsDNA virus, and the replication of its viral genome leads to accumulation of dsRNAs, which are recognized by endosomal TLR3 (Piret and Boivin, 2015). The TLR-like receptor-associated interferon (IFN) factor (TRIF) signaling pathway activates mitogen-activated protein kinase (MAPK), which induces the production of pro-inflammatory cytokines. On the other hand, nuclear transcription of NF-κB is induced by NF-κB essential modulator (NEMO), which stimulates the production of pro-inflammatory cytokines such as TNF-α, ILs, and type I IFN. At the same time, TNF receptor-associated factor 3 (TRAF3) is activated through the IFN regulatory factor 3 (IRF3) pathway to stimulate the production of type I IFN (Kawai and Akira, 2011; Kong and Le, 2011; Moresco et al., 2011). Pro-inflammatory cytokines are essential to the host’s antiviral response. However, in excess, they can cause tissue damage and related pathological changes (Carty and Bowie, 2011; Zhang et al., 2013).

Therefore, the aim of this study was to investigate the molecular mechanism *via* which corilagin controls the immune response and inflammatory injury in HSE through the TLR3 signaling pathway.

## Materials and Methods

### Reagents

Corilagin standard substance with purity >98% was purchased from Yuanye Biotechnology Co., Ltd (B-20672, Shanghai, China). High-glucose Dulbecco’s Modified Eagle’s Medium (DMEM, SH30022.01) and phosphate-buffered saline (PBS, SH30256.01) buffer solution were purchased from HyClone (Logan, UT, USA). Fetal bovine serum (FBS) was offered by Zhejiang Tianhang Biotechnology Co., Ltd (11011-8611, Hangzhou, China). Trypsin 0.25% with EDTA was purchased from Genom Biotechnology Co., Ltd (GNM25200, Hangzhou, China). Dimethyl sulfoxide (DMSO) was purchased from Sigma-Aldrich China (D2650, Shanghai, China). Cell counting kit-8 (CCK-8) was purchased from Dojindo Company (CK04, Mashikimachi, Japan). Polyriboinosinic polyribocytidylic acid [poly(I:C)] was purchased from InvivoGen (tlrl-picw, San Diego, CA, USA). TNF-α (E-EL-M0049c) and IL-6 (E-EL-M0044c) enzyme-linked immunosorbent assay (ELISA) kits were purchased from Elabscience Biotechnology Co., Limited (Wuhan, China). Total RNA Extraction Reagent (RNAiso Plus, 9108), 5× Prime Script^®^ RT Master Mix Reverse Transcription Kit (RR036Q), and SYBR^®^Premix Ex TaqTM II (Perfect Real Time, RR420A) were purchased from TaKaRa (Dalian, China). Phenylmethylsulfonyl fluoride (PMSF, ST506), Radio Immunoprecipitation Assay (RIPA) Lysis and Extraction Buffer (P0013B), and Enhanced BCA Protein Assay Kit (P0012) were bought from Beyotime (Shanghai, China). Rabbit anti-mouse TLR3 (DF6415), TRIF(DF6289), TNF receptor type 1-associated DEATH domain protein (TRADD, DF6279), TRAF3 (DF7181), TRAF6 (AF5376), P38(AF6456), NEMO (DF6143), and IRF3 (DF6895) antibodies were purchased from Affinity (Cincinnati, OH, USA). Horseradish peroxidase (HRP)-labeled goat anti-rabbit immunoglobulin G (IgG) was purchased from Boster Immunoleader (BA1054, Fremont, CA, USA). The electrochemiluminescence (ECL) kit was provided by Millipore (WBKLS0100, Darmstadt, Germany). All primers were synthesized by Tsingke Biological Technology (Wuhan, China).

### Cell and Virus Culture

The HSV-1 virus (central lab of Wuhan Union Hospital) was cultured and proliferated with HeLa cells. Mouse BV-2 microglia [China Center for Type Culture Collection (Wuhan, China)] was used as a cellular model *in vitro*. The cells were cultured in an incubator at 37°C, 5% CO_2_, and 95% humidified air with high-glucose DMEM containing 10% FBS. Various virus dilutions were inoculated into BV-2 cells. The virus infectivity of each virus dilution was determined by the cytopathic effect (CPE) method, and the titer of the virus (CCID_50_) was calculated by the Reed–Muench method.

### Corilagin-Induced Cytotoxicity

The CCK8 assay was used to study corilagin-induced cytotoxicity as described previously (Wang et al., 2017). The cell suspension (5 × 10^4^ cells/mL) was added to a 96-well plate and the cells were cultured for 24 h. Different concentrations of corilagin were added to the 96-well plate in triplicate. After 24 h, changes in cell morphology were observed under a regular phase-contrast microscope. Then, DMEM with 10 μL CCK-8 solution was added to each well and the cells were placed in the incubator for 1–4 h. The absorbance at 450 nm was measured at multiple time points on a microplate reader.

### Establishment of a Cellular Model and Treatment With Corilagin

Poly(I:C), an agonist of TLR3, served as a positive control same as the HSV-1 group. Cells incubated with DMEM were defined as the normal group (normal means unstimulated BV2 cells group). BV2 cells were moved to the plate overnight. The supernatants of adhered cells were removed, and poly(I:C; 10 μg/mL) or HSV-1 (100TCID_50_, TCID_50_ = 10^−2.67^/0.1 mL) were added to the wells. After a 6 h incubation, the culture medium was changed and the cells were treated with corilagin (the concentration is shown in the “Results” section) or ACV (40 μg/mL) for 24 h.

### Lentiviral Vector-Mediated Overexpression of TLR3 in BV-2 Cells

The procedure was conducted as described previously by our group (Jin et al., 2017). Lentiviral vectors for TLR3 and a negative control were constructed by Tuojie (Wuhan, China). The lentiviral vector was transfected into the 293T cell line and the virus supernatant was collected 48 h later [1 × 10^8^ transducing units (TU)/mL]. The BV-2 cells were transfected with lentivirus at a multiplicity of infection (MOI) of 10 according to the experimental guidance. The medium was disposed 6 h later, and the cells were incubated for 72 h. TLR3 overexpression was confirmed by real-time polymerase chain reaction (PCR).

### siRNA-TLR3-Mediated Down-Regulation of TLR3 in BV-2 Cells

TLR3 siRNA (sense 5′-TCTGGAGTACAACAATATA-3′) and a negative control were synthesized by Guangzhou Ribo Biotechnology Co., Limited (Guangzhou, China). BV-2 cells were seeded in six-well plates and transfected with 50 nM siRNA using Lipofectamine^®^ 2000 liposome transfection agent (Invitrogen, San Diego, CA, USA) according to the manufacturer’s instructions. After 6 h, the medium was changed and the cells were cultured for another 24 h. TLR3 down-regulation was confirmed by real-time PCR.

### Animals

Three-week-old male Balb/c mice weighing 11–13 g were purchased at the Experimental Animal Center of Tongji Medical College, Huazhong University of Science and Technology (Wuhan China). For the experiments, we first injected 20 μL of virus solution at various dilutions in DMEM directly into the brain at the midpoint of the line from the right canthus to the external auditory canal. The mortality rate was recorded for each group and the median lethal dose (LD_50_) was calculated using the Reed-Muench method. Then, 40 male Balb/c mice were randomly divided into four groups: two normal groups (the DMEM group, 20 μL per mouse, *n* = 5; and the PBS group, 20 μL per mouse, *n* = 5) and two model groups [the poly(I:C) group, 5 mg/kg, *n* = 15; and the HSV-1 group, 20 μL LD_50_ virus suspension per mouse, *n* = 15]. The mice were anesthetized by intraperitoneal injection of 10% chloral hydrate (3.5 mL/kg of body weight). Next, DMEM, PBS, poly(I:C), or HSV-1 were injected into the intracalvarium at the midpoint of the line from the right canthus to the external auditory canal. One hour after the model was established, the normal groups were given normal saline (NS) intragastrically; five mice in each model group were given NS, corilagin 40 mg/kg or ACV 350 mg/kg daily intragastrically. On day 5, mice were sacrificed and the right temporal lobe brain tissues were dissected.

### Enzyme-Linked Immunosorbent Assay (ELISA) for IL-6, TNF-α, and IFN-β

The levels of IL-6, TNF-α, and IFN-β in the cell supernatants and brain tissues were determined by ELISA according to the manufacturer’s instructions.

### Real-Time Quantitative PCR Analysis

Total RNA in BV-2 cells and brain tissues was extracted using RNAiso Plus. The RNA was reverse-transcribed into cDNA using the PrimeScript RT Kit and incubated at 37°C for 15 min and 85°C for 5 s. The StepOne Plus device (Applied Biosystems) was used to perform real-time PCR at 95°C for 10 s followed by 40 cycles at 95°C for 5 s and 60°C for 20 s according to the instructions of the SYBR Premix Ex Taq kit. The data were analyzed by the 2^−ΔΔCt^ method. All primers were synthesized by TSINGKE (Wuhan, China). The primer sequences were as follows:

- TLR3: forward, GATACAGGGATTGCACCCATA; reverse, TCCCCCAAAGGAGTACATTAGA
- TRIF: forward, GCAGAGTCGGGGTAACAAGA; reverse, CCAGAAGGTGGTGCTCAAATA
- TRADD: forward, GTTCGAAGTTCCCGGTTTCC; reverse, CTCTCAGTGCCCGACAGTTA
- TRAF3: forward, TCAGGTCTACTGTCGGAATGAA; reverse, ATCCCGCAAGTCTTTTCTCAG
- TRAF6: forward, AAACCACGAAGAGGTCATGG; reverse, GCGGGTAGAGACTTCACAGC
- NEMO: forward, GGTGGAGAGACTGAGCTTGG; reverse, CTAAAGCTTGCCGATCCTTG
- P38: forward, ATCATTCACGCCAAAAGGAC; reverse, AGCTTCTGGCACTTCACGAT
- IRF3: forward, CACTCTGTGGTTCTGCATGG; reverse, ATGTCCTCCACCAAGTCCTG
- GAPDH: forward, CAGCAAGGACACTGAGCAAGA; reverse, GCCCCTCCTGTTATTATGGGG

#### Western Blot Analysis

Following our previous procedures (Yang et al., 2016), total protein was extracted from BV-2 cells and brain tissues using RIPA Lysis and Extraction Buffer. Protein concentrations were determined by using a bicinchoninic acid (BCA) kit. Proteins were separated by sodium dodecyl sulfate polyacrylamide gel electrophoresis (SDS-PAGE) for approximately 90 min before being transferred to polyvinylidene difluoride (PVDF) membranes. The membranes were blocked with 5% skim milk dissolved in Tris-buffered saline with Tween 20 (TBST) at room temperature for 1 h and probed with antibodies against TLR3, TRIF, TRADD, TRAF6, TRAF3, P38, NEMO, and IRF3 (1:1,000) overnight at 4°C. The next day, membranes were washed three times in TBST and then incubated with the corresponding HRP-labeled secondary antibodies (1:20,000). After washing the membranes three times with TBST, ECL reagent was used to identify immunoreactive bands. The signals were detected by the Fuji Ultrasonic-Doppler Velocity Profile (UVP) system and analyzed using Image J.

#### Hematoxylin-Eosin (HE) Staining

The procedures were conducted as described previously (Jin et al., 2015; Ding et al., 2016). The right temporal lobe brain tissues were dissected and fixed in 4% paraformaldehyde. After being embedded in paraffin and cut into slices, the brain tissue specimens were stained with HE for observing the histological changes.

#### Immunohistochemistry (IHC)

IHC was performed as described previously (Yang et al., 2017). Brain tissue specimens were cut into 10-μm sections before dewaxing and moisturizing. Then, sections were incubated in 3% H_2_O_2_/methanol to eliminate endogenous peroxidase activity. Afterward, sections were incubated with normal goat serum for 20 min and then incubated with TLR3 antibody (1:400) overnight at 4°C. After incubation, the sections were washed with PBS and incubated with biotinylated secondary antibody for 45 min at 37°C. They were rinsed with PBS again and incubated with streptavidin–biotin HRP complex (SABC) at 37°C. Samples were stained with diaminobenzidine (DAB) and hematoxylin. After slides were rinsed with distilled water and dehydrated, they were made transparent and mounted for microscopic examination. IPP software (Image-Pro Plus 6.0) was used to analyze the images of immunohistochemical slides.

#### Statistical Analysis

Data are expressed as mean ± standard deviation (SD). Comparisons between two groups were performed using Student’s *t*-test. For comparisons of multiple groups and all other statistical analyses, one-way analysis (ANOVA) was used, followed by Tukey’s *post hoc* test. A *P*-value less than 0.05 or 0.01 was considered statistically significant (Dang et al., 2017). The statistical analyses were performed using SPSS software and the graphs were drawn on GraphPad Prism 6.0 (GraphPad Software, La Jolla, CA, USA; Ding et al., 2015).

## Results

### Corilagin Induced Cytotoxicity in BV-2 Cells

**Figure 1:**
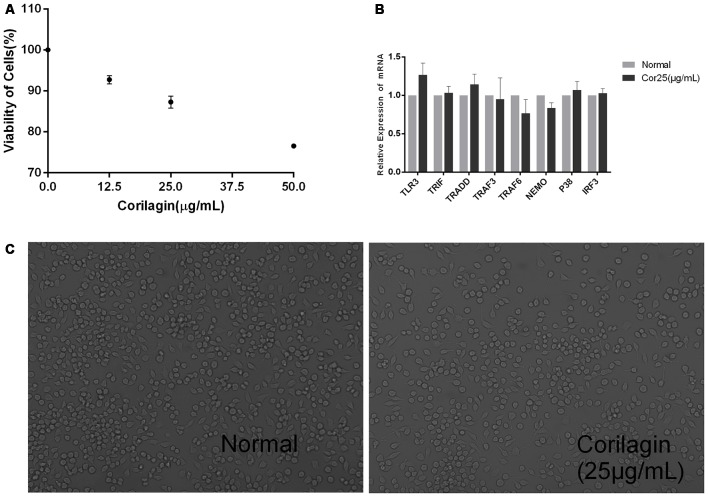
Corilagin induced cytotoxicity in BV-2 cells. Cells were cultured with different concentrations of corilagin for 24 h. **(A)** BV-2 cells were cultured with corilagin (12.5, 25, or 50 μg/mL) for 24 h, and cell viability was determined by the cell counting kit-8 (CCK-8) assay. **(B)** Total RNA was extracted, and the mRNA levels of toll-like receptor 3 (TLR3) signaling components were measured by real-time quantitative polymerase chain reaction (PCR). Data are shown as the mean ± SD (*n* = 3), *P* > 0.05 (by Student’s *t*-test). **(C)** BV-2 cell morphology was observed under a regular phase-contrast microscope after treatment with corilagin (25 μg/mL) for 24 h (scale bar: 100 μm).

Unstimulated BV-2 cells were cultured with different concentrations of corilagin (12.5, 25, or 50 μg/mL) for 24 h. The results of the CCK-8 assay showed that cell viability was higher than 87% at the concentration of 25 μg/mL (Figure 1A). RT-PCR and cell morphology analyses showed no significant differences between the normal group and the corilagin-treated group (Figures 1B,C). Therefore, in further experiments, the cells were treated with 25 μg/mL corilagin for 24 h.

### Poly(I:C) and HSV-1 Activated the TLR3 Signaling Pathway in BV-2 Cells

**Figure 2:**
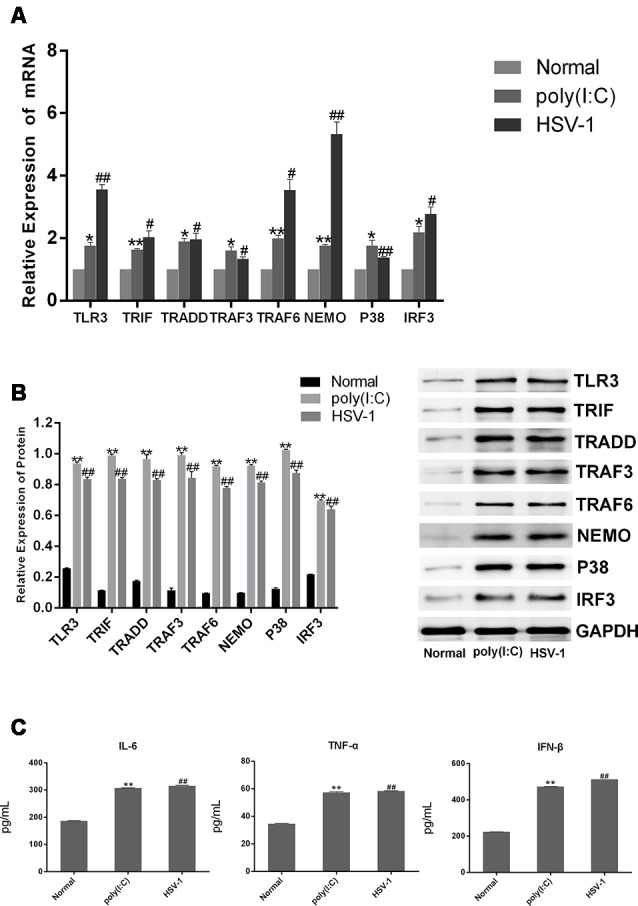
Poly(I:C) and herpes simplex virus (HSV)-1 increased the expression of TLR3 signaling components in BV-2 cells. BV2 cells were stimulated by poly(I:C; 10 μg/mL) and HSV-1 (100TCID50, 10^−2.67^/0.1 mL) for 6 h, then the fresh medium was added in for culturing another 24 h. **(A)** The total RNA was extracted from activated BV-2 cells. The mRNA levels of TLR3 and its downstream molecules were measured by RT-PCR. **(B)** The protein levels of TLR3 and its downstream molecules were detected bywestern blotting. **(C)** The levels of tumor necrosis factor (TNF)-α, interleukin-6 (IL-6), and interferon (IFN)-β in the supernatant were measured by enzyme-linked immunosorbent assay (ELISA). Data are shown as the mean ± SD of three independent experiments of triplicate samples. *P < 0.05, **P < 0.01 for poly(I:C) vs. normal; ^#^*P* < 0.05, ^##^*P* < 0.01 for HSV-1 vs. normal (by Student’s *t*-test).

To confirm that HSV-1 activates the TLR3 signaling pathway in BV-2 cells, we cultured BV-2 cells with poly(I:C) or HSV-1 for 24 h and detected TLR3 and its downstream molecules by RT-PCR and western blotting. The mRNA expression of TLR3, TRIF, TRADD, TRAF3, TRAF6, NEMO, P38, and IRF3 were obviously increased about two-fold in the cells stimulated with poly(I:C) or HSV-1 compared with the normal group (Figure 2A), meanwhile the protein expression of the molecules were increased about four-fold (Figure 2B). The secretion of pro-inflammatory cytokines such as IL-6, TNF-α, and IFN-β were also significantly increased about two-folds compared with the normal group (Figure 2C). Therefore, the TLR3 pathway was activated by poly(I:C) and HSV-1.

### Corilagin Interfered With the TLR3 Signaling Pathway in BV-2 Cells Stimulated by Poly(I:C) or HSV-1

**Figure 3:**
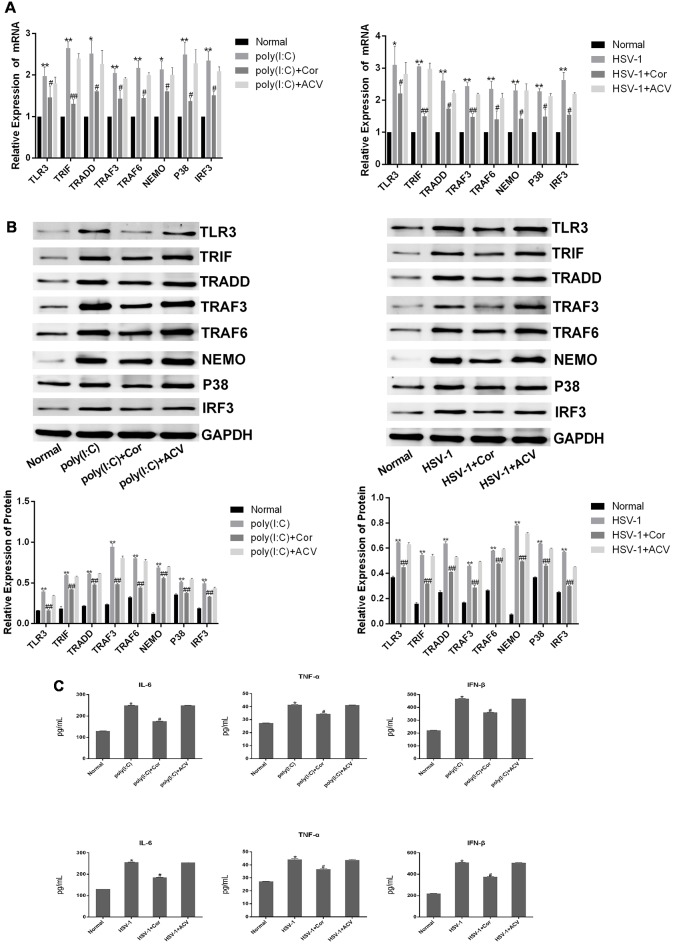
Corilagin interfered with the TLR3 signaling pathway in BV-2 cells stimulated by poly (I:C) or HSV-1. BV2 cells were co-cultured with poly(I:C; 10 μg/mL) and HSV-1 (100TCID50, 10^−2.67^/0.1 mL) for 6 h, and then the supernatants were removed. BV2 cells were treated with Corilagin (25 μg/mL) or acyclovir (ACV; 40 μg/mL) for 24 h. **(A)** The mRNA levels of TLR3 and its downstream molecules were measured by RT-PCR. **(B)** The protein levels of TLR3 and its downstream molecules were detected by western blotting. **(C)** The levels of TNF-α, IL-6, and IFN-β in the supernatant were measured by ELISA. Data are shown as the mean ± SD of three independent experiments of triplicate samples. *P < 0.05, **P < 0.01 for poly (I:C) or HSV-1 vs. normal; ^#^*P* < 0.05, ^##^*P* < 0.01 for poly (I:C) or HSV-1+corilagin 25 μg/mL vs. poly (I:C) or HSV-1 (by Student’s *t*-test).

To investigate whether corilagin affects the expression of TLR3 and its downstream molecules, we used poly(I:C) and HSV-1 to activate the TLR3 signaling pathway. After BV-2 cells were treated with corilagin, the mRNA levels of TLR3, TRIF, TRADD, TRAF3, TRAF6, NEMO, P38, and IRF3 were reduced about 30% compared with the poly(I:C) or HSV-1 group (Figure 3A). Similar reductions were observed in protein expression (Figure 3B). Furthermore, the secretion of IL-6, TNF-α, and IFN-β in corilagin-treated cells was about 30% lower than in the poly(I:C) and HSV-1 groups (Figure 3C). However, there were no significant changes between the ACV group and the poly(I:C) or the HSV-1 group.

### Corilagin Interfered With the TLR3 Signaling Pathway in BV-2 Cells Overexpressing TLR3

**Figure 4:**
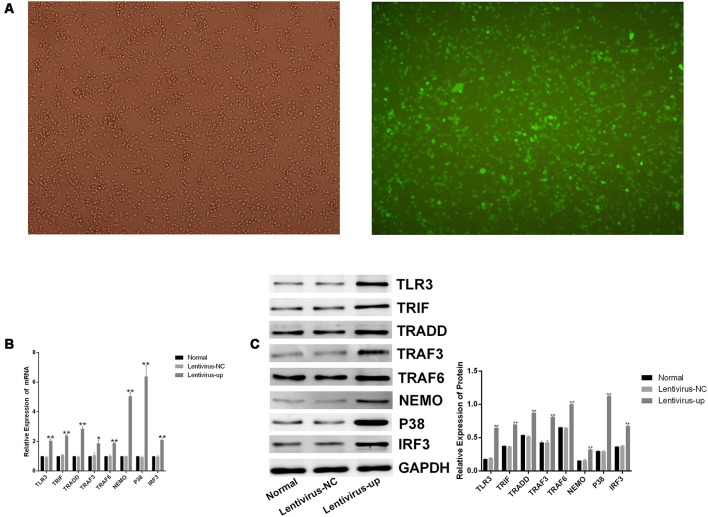
Lentiviral vector-mediated overexpression of TLR3 in BV-2 cells. The lentivirus (10 MOI) was incubated with the BV-2 cells for 72 h. **(A)** The expression of green fluorescent protein (GFP) was observed with a fluorescence microscope after lentivirus was introduced into the BV-2 cells. **(B)** The mRNA levels of TLR3 and its downstream molecules were measured by RT-PCR. **(C)** The protein levels were detected by western blotting. Data are shown as the mean ± SD of three independent experiments of triplicate samples. **P < 0.01 for lentivirus-up vs. normal; *P < 0.05 for lentivirus-up vs. normal (by Student’s *t*-test).

**Figure 5:**
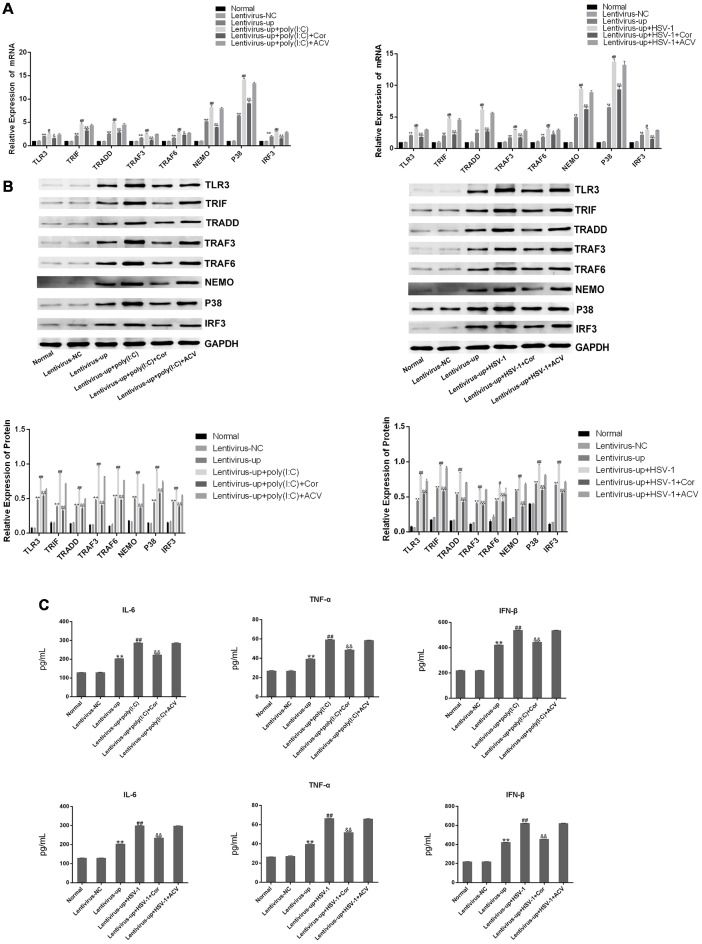
Corilagin interfered with the TLR3 signaling pathway in BV-2 cells overexpressing TLR3. The lentivirus vectors (10MOI) were transfected into BV2 cells for 72 h, and then the poly(I:C; 10 μg/mL) and HSV-1 (100TCID50, 10^−2.67^/0.1 mL) were added into cells for stimulating 6 h. Then the supernatants were replaced, and cells were treated with Corilagin (25 μg/mL) or ACV (40 μg/mL) for 24 h. **(A)** The mRNA levels of TLR3 and its downstream molecules were detected by RT-PCR. **(B)** The protein levels of TLR3 and its downstream molecules were measured by western blotting. **(C)** The levels of TNF-α, IL-6, and IFN-β in the supernatant were measured by ELISA. Data are shown as the mean ± SD of three independent experiments of triplicate samples. **P < 0.01 for lentivirus-up vs. normal, ^#^*P* < 0.05, ^##^*P* < 0.01 for lentivirus-up+poly (I:C) or HSV-1 vs. lentivirus-up, ^&^*P* < 0.05, ^&&^*P* < 0.01 for lentivirus-up+poly (I:C) or HSV-1+Cor vs. lentivirus-up+poly (I:C) or HSV-1 (by Student’s *t*-test).

To confirm the efficacy of corilagin, a lentiviral vector encoding TLR3 was transfected into BV-2 cells for 72 h. Green fluorescent protein (GFP) was observed with a fluorescence microscope (Figure 4A). RT-PCR and western blotting revealed that TLR3 and its downstream molecules were overexpressed (Figures 4B,C). BV-2 cells were stimulated with poly(I:C) or HSV-1 for 6 h, after which corilagin or ACV was added to cells for 24 h before harvesting. Compared with the lentivirus-up+poly(I:C) or the HSV-1 group, the mRNA (Figure 5A) levels of TLR3, TRIF, TRADD, TRAF3, TRAF6, NEMO, P38, and IRF3 were decreased 20%–50% in the lentivirus-up+poly(I:C) or the HSV-1+Cor group, meanwhile protein (Figure 5B) levels were reduced 30%–60%. However, compared with the lentivirus-up+poly(I:C) or the HSV-1 group, the mRNA and protein levels of the lentivirus-up+poly(I:C) group or the HSV-1+ACV group were not significantly reduced. The levels of IL-6, TNF-α, and IFN-β in the lentivirus-up+poly(I:C) group or the HSV-1+Cor group were 20% lower than in the lentivirus-up+poly(I:C) or the HSV-1 group (Figure 5C).

### Corilagin Interfered With the TLR3 Signaling Pathway in BV-2 Cells in Which TLR3 Was Silenced

**Figure 6:**
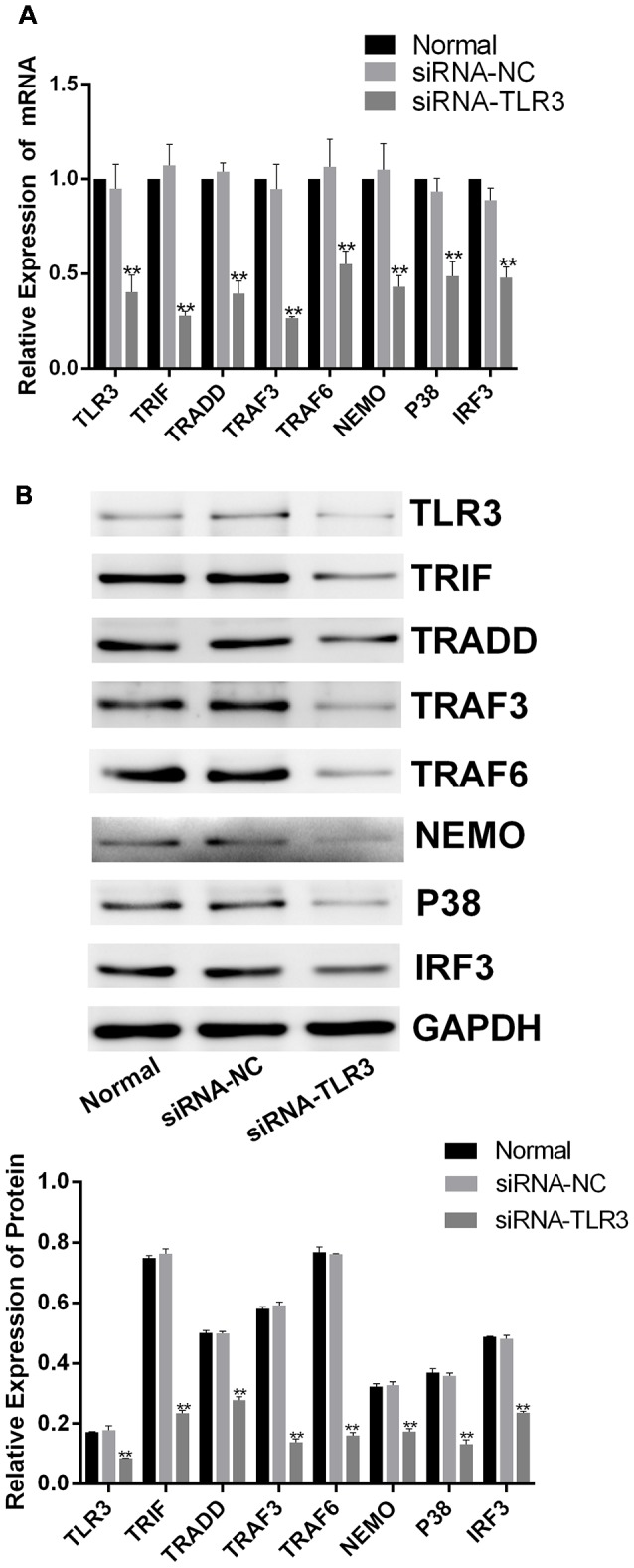
siRNA-TLR3-mediated down-regulation of TLR3 in BV-2 cells. The siRNAs (50 nM) were transfected into BV-2 cells by using Lipofectamine^®^ 2000 liposome for 6 h. Then, the supernatants were replaced, and the cells were cultured for another 24 h. **(A)** The mRNA levels of TLR3 and its downstream molecules were detected by RT-PCR. **(B)** The protein levels of TLR3 and its downstream molecules were measured by western blotting. Data are shown as the mean ± SD of three independent experiments of triplicate samples. **P < 0.01 for siRNA vs. normal (by Student’s *t*-test).

**Figure 7:**
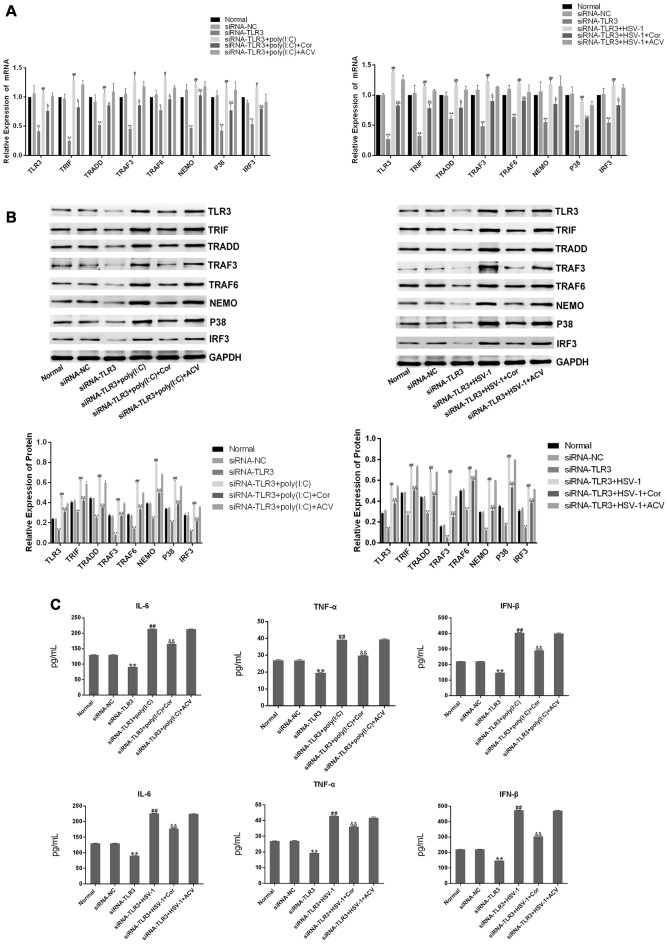
Corilagin interfered with the TLR3 signaling pathway in BV-2 cells in which TLR3 was silenced. After siRNA (50 nM) was transfected into BV2 cells, then the poly(I:C; 10 μg/mL) and HSV-1 (100TCID50, 10^−2.67^/0.1 mL) were added into cells for stimulating 6 h. Meanwhile, the supernatants were replaced, and cells were treated with Corilagin (25 μg/mL) or ACV (40 μg/mL) for 24 h.** (A)** The mRNA levels of TLR3 and its downstream molecules were detected by RT-PCR. **(B)** The protein levels of TLR3 and its downstream molecules were measured by western blotting.** (C)** The levels of TNF-α, IL-6, and IFN-β in the supernatant were measured by ELISA. Data are shown as the mean ± SD of three independent experiments of triplicate samples. *P < 0.05, **P < 0.01 for siRNA-TLR3 vs. normal, ^#^*P* < 0.05, ^##^*P* < 0.01 for siRNA-TLR3+poly(I:C) or HSV-1 vs. siRNA-TLR3, ^&^*P* < 0.05, ^&&^*P* < 0.01 for siRNA-TLR3+poly(I:C) or HSV-1+Cor vs. siRNA-TLR3+ poly(I:C) or HSV-1 (by Student’s *t*-test).

To further confirm the efficacy of corilagin, siRNAs (50 nM) were transfected into BV-2 cells to silence the expression of TLR3 using Lipofectamine^®^ 2000. To confirm the efficiency of transduction, the mRNA and proteins levels of TLR3 and its downstream molecules were determined after transfection (Figures 6A,B); and expression levels are reduced by about 50%. BV-2 cells were then stimulated with poly(I:C) or HSV-1 for 6 h, after which the supernatant was changed. Then, the cells were treated with corilagin or ACV for 24 h. The mRNA levels of TLR3, TRIF, TRADD, TRAF3, TRAF6, NEMO, P38, and IRF3 were decreased 20%–30%, compared with siRNA-TLR3+poly(I:C) or HSV-1, at the same time, the protein levels were decreased 20%–40%; siRNA-TLR3+poly(I:C) or HSV-1+ACV did not result in significant changes (Figures 7A,B). Compared with the siRNA-TLR3+poly(I:C) or HSV-1, the secretion of IL-6, TNF-α, and IFN-β was decreased 15%-35% after treated with Corilagin (Figure 7C).

### Corilagin Induces Histopathological Changes in the Brain of Mice With Encephalitis

**Figure 8:**
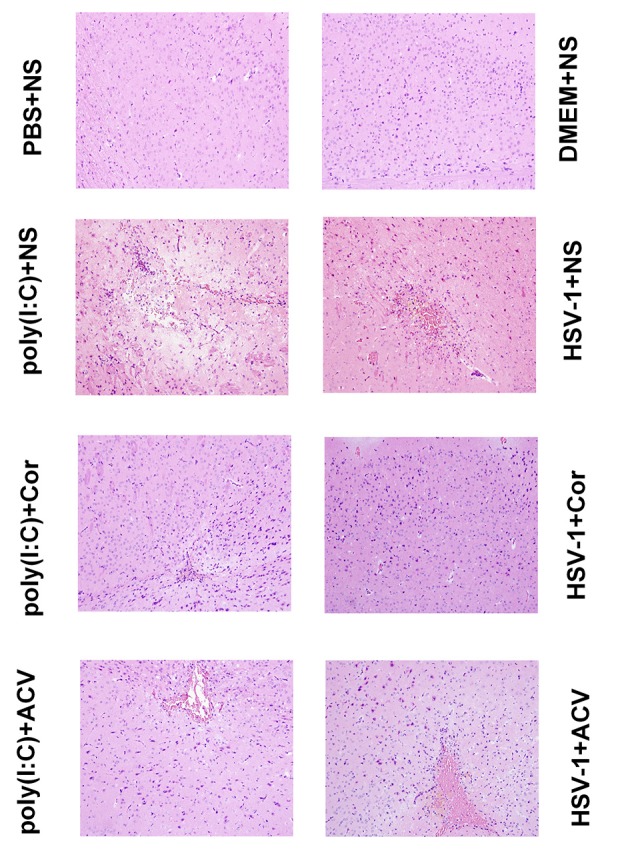
Corilagin induces histopathological changes in the brain of mice with encephalitis. Histopathological changes in the right temporal lobe brain tissue were observed by hematoxylin-eosin (HE) staining (×200). DMEM (20 μL), phosphate-buffered saline (PBS; 20 μL), poly(I:C; 5 mg/kg, 20 μL), or HSV-1 (10^−4^/20 μL) were injected into the intracalvarium at the midpoint of the line from the right canthus to the external auditory canal. One hour after the model was established, five mice in each group were given normal saline (NS), corilagin 40 mg/kg, or ACV 350 mg/kg daily intragastrically. The brain tissues were taken for HE staining.

The median lethal dose (LD_50_) of the virus (10^−4^/20 μL) was calculated by the improved Karber’s method. The mice became sick the next day after they were injected with HSV-1 virus (10^−4^/20 μL) and poly(I:C; 5 mg/kg). The symptoms included piloerection, apathy, crouching, hemiplegia, and convulsion. The PBS+NS and DMEM+NS group survived at the end of the 5th day. The survival rate of the poly(I:C)+NS group was 40%, the HSV-1+NS group was 20%. After treatment with Corilagin, the poly(I:C)+Cor group survived, and the survival rate of HSV-1+Cor group was 80%. Then mice were sacrificed, and the right temporal lobe brain tissues were taken for HE staining (Figure 8). HE analysis revealed that the PBS or the DMEM+NS group had complete cell structure without obvious inflammatory infiltration. Compared with the PBS or the DMEM+NS group, the poly(I:C) and the HSV-1+NS groups showed infiltration of inflammatory cells, a necrotic temporal lobe, and large areas of hemorrhage. In the poly(I:C) and the HSV-1+Cor groups, the pathological changes were not as obvious as in the poly(I:C) and the HSV-1+NS groups. However, the pathological changes in the poly(I:C) and the HSV-1+ACV group were not as great as those in the poly(I:C) and the HSV-1+NS groups.

### Corilagin Reduces TLR3 Protein Expression in the Brains of Mice With Encephalitis

**Figure 9:**
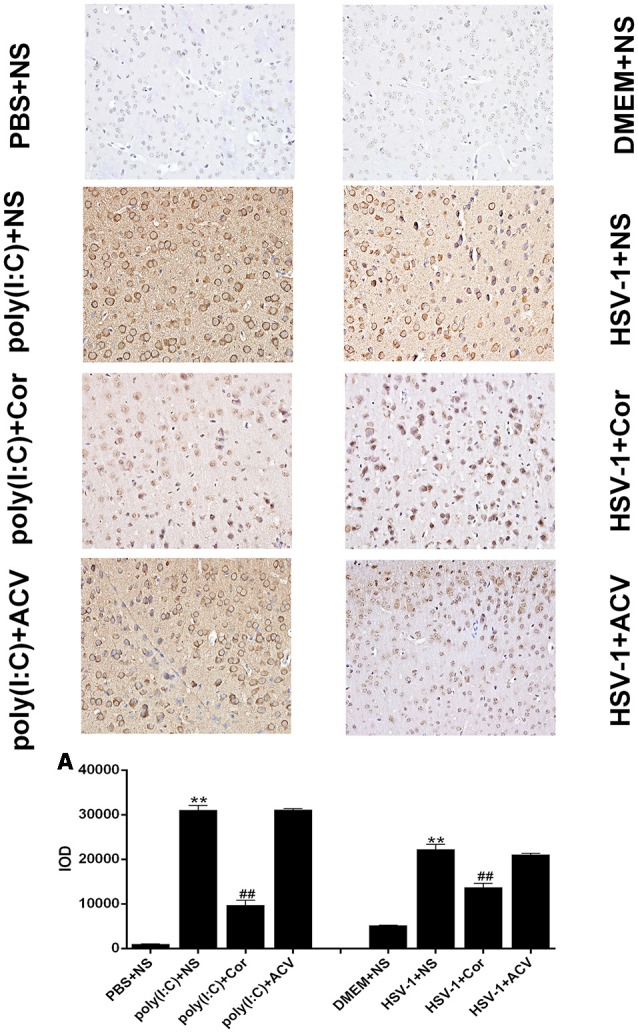
Corilagin reduces TLR3 protein expression in the brains of mice with encephalitis. The TLR3 protein levels in right temporal lobe brain tissue were analyzed by immunohistochemistry (IHC; ×400). DMEM (20 μL), PBS (20 μL), poly(I:C; 5 mg/kg, 20 μL), and HSV-1 (10^−4^/20 μL) were injected into the intracalvarium at the midpoint of the line from the right canthus to the external auditory canal. After the model was established for 1 h, five mice in each group were intragastrically treated with NS, Corilagin 40 mg/kg, ACV 350 mg/kg each day. The brain tissues were taken for IHC analysis.** (A)** The TLR3 protein levels in the right temporal lobe brain were detected by IHC and the optical density of the image was analyzed by IPP software. Data are shown as the mean ± SD of three independent experiments of triplicate samples. **P < 0.01 for poly(I:C) or HSV-1+NS vs. PBS or DMEM+NS, ^##^*P* < 0.01 for poly(I:C) or HSV-1+Cor vs. poly(I:C) or HSV-1+NS (by Student’s *t*-test).

To investigate whether corilagin inhibits the expression of TLR3 to relieve brain inflammation, we performed IHC analysis of TLR3 in the brain (Figure 9). The positive staining of TLR3 was dark brown. The staining of TLR3 in the PBS and the DMEM+NS groups was not clearly visible, whereas that in the HSV-1 and poly(I:C)-injected mice was significantly increased. After corilagin treatment, the expression of TLR3 was prominently decreased and the staining was lighter than that in the poly (I:C) or the HSV-1+NS group. However, the positive staining rate in the poly (I:C) or the HSV-1+ACV group showed no remarkable differences compared with the poly (I:C) or the HSV-1+NS group.

### Corilagin Interferes With the TLR3 Signaling Pathway in the Brain of Mice With Encephalitis

**Figure 10:**
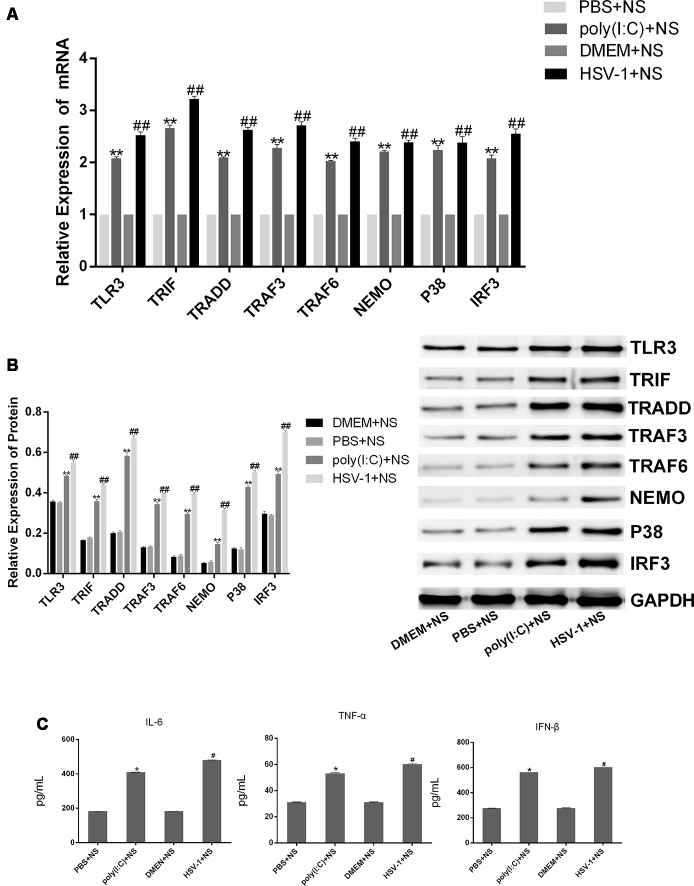
The expression of TLR3 and its downstream molecules in brain tissues of mice with encephalitis. DMEM (20 μL), PBS (20 μL), poly(I:C; 5 mg/kg, 20 μL), and HSV-1 (10^−4^/20 μL) were injected into the intracalvarium at the midpoint of the line from the right canthus to the external auditory canal. After the model was established for 1 h, five mice in each group were intragastrically treated with NS, Corilagin 40 mg/kg, ACV 350 mg/kg each day.** (A)** The mRNA levels of TLR3 and its downstream molecules in brain tissues were detected by RT-PCR. **(B)** The protein levels of TLR3 and its downstream molecules in brain tissues were measured by western blotting.** (C)** The levels of TNF-α, IL-6, and IFN-β in brain tissues were measured by ELISA. Data are shown as the mean ± SD of three independent experiments of triplicate samples. *P < 0.05, **P < 0.01 for poly(I:C)+NS vs. PBS+NS, ^#^*P* < 0.05, ^##^*P* < 0.01 for HSV-1+NS vs. DMEM+NS (by Student’s *t*-test).

**Figure 11:**
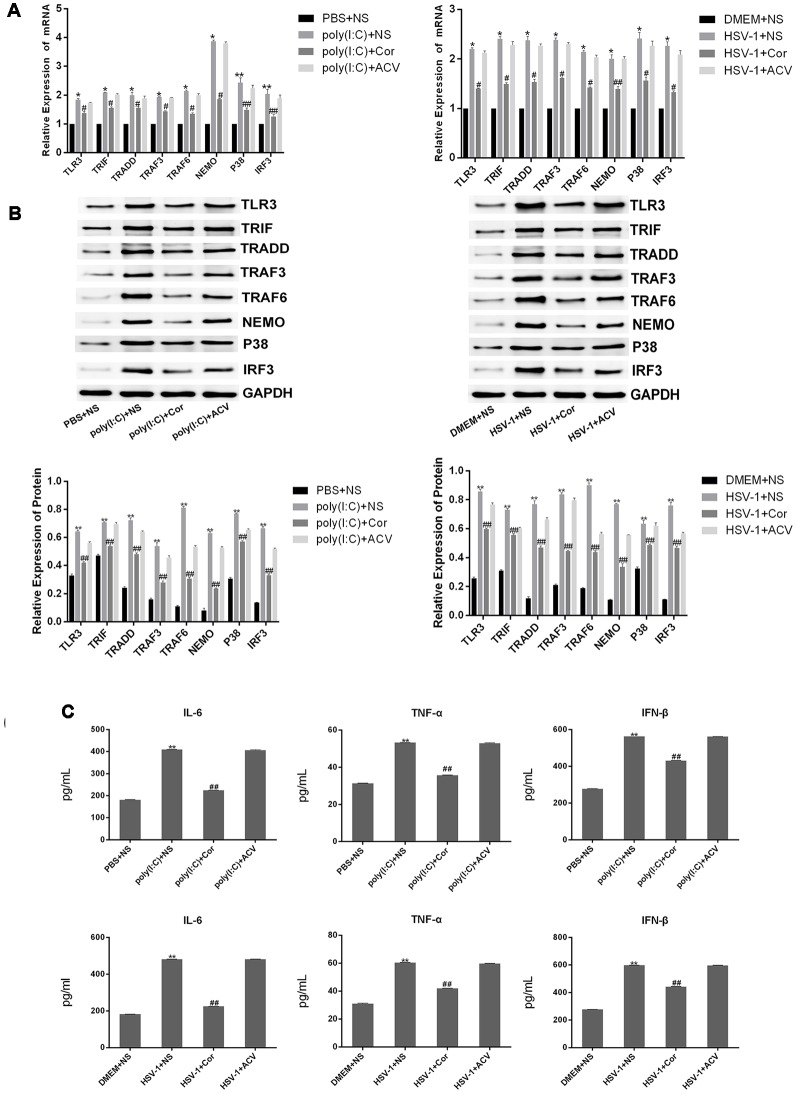
Corilagin interferes with the TLR3 signaling pathway in the brain of mice with encephalitis. DMEM (20 μL), PBS (20 μL), poly(I:C) (5 mg/kg, 20 μL), and HSV-1 (10^−4^/20 μL) were injected into the intracalvarium at the midpoint of the line from the right canthus to the external auditory canal. After the model was established for 1 h, five mice in each group were intragastrically treated with NS, Corilagin 40 mg/kg, ACV 350 mg/kg each day. The brain tissues were taken for test. **(A)** The mRNA levels of TLR3 and its downstream molecules in brain tissues were detected by RT-PCR. **(B)** The protein levels of TLR3 and its downstream molecules in brain tissues were measured by western blotting.** (C)** The levels of TNF-α, IL-6, and IFN-β in brain tissues were measured by ELISA. Data are shown as the mean ± SD of three independent experiments of triplicate samples. *P < 0.05, **P < 0.01 for poly(I:C)+NS vs. PBS+NS, ^#^*P* < 0.05, ^##^*P* < 0.01 for HSV-1+NS vs. DMEM+NS (by Student’s *t*-test).

To investigate whether corilagin inhibits the TLR3 signaling pathway to relieve the inflammatory response, we examined the expression of TLR3 and its downstream molecules in the brain of mice with encephalitis. The mRNA and protein expression of TLR3, TRIF, TRADD, TRAF3, TRAF6, NEMO, P38, and IRF3 as well as the secretion of IL-6, TNF-α, and IFN-β in the poly(I:C) and the HSV-1+NS groups were two-folds higher than in the PBS and the DMEM+NS groups, suggesting that the TLR3 pathway was activated after mice were infected with poly(I:C) or HSV-1 (Figure 10). After administration of corilagin, the mRNA levels of TLR3 and its downstream molecules were reduced 20%–40% (Figure 11A). The protein levels of TLR3, TRIF, TRADD, TRAF3, TRAF6, NEMO, P38, and IRF3 were decreased 20%–60% compared with the poly(I:C) and the HSV-1+NS groups (Figure 11B). The secretion of IL-6, TNF-α, and IFN-β in brain tissue, as measured by ELISA, was decreased 30%–50% compared with the poly(I:C) and the HSV-1+NS groups (Figure 11C). However, these molecules were not significantly decreased in the poly(I:C) and the HSV-1+ACV groups.

## Discussion

HSE is a common form of sporadic encephalitis that lacks curative treatment. Almost all adult HSE cases are caused by HSV-1 (Whitley, 2006). HSE causes acute inflammation, brain tissue edema, softened, hemorrhage, and necrosis. Both cerebral hemispheres are involved, most notably the frontal and temporal lobes, usually asymmetrically (Whitley and Lakeman, 1995; Whitley, 2006). Unfortunately, the pathogenesis of HSE has remained elusive. Some studies proposed TLRs as the PRRs that recognize HSV-1 proteins or viral nucleic acids to initiate the innate immune response (Medzhitov and Janeway, 2002), aggravating the condition of HSE. Studies have shown that the release of large amounts of pro-inflammatory cytokines, mediated by TLR2, TLR3, and TLR9 signaling pathways, is the main cause of HSE brain tissue damage (Conrady et al., 2010).

Previous studies have shown that the inhibitory target of corilagin in the TIRAP/MyD88-TRAF6 pathway was cell-surface TLR2 (Guo et al., 2015a,b). HSV-1 is an enveloped double-stranded DNA virus with neurotropic properties that belongs to the alpha herpesviridae family. In the process of HSV-1 replication, dsRNA can be recognized by endosomal TLR3 (Boehme and Compton, 2004; Conrady et al., 2010), which activates the TLR3 signaling pathway. The activation of TLR3 recruits TRIF and stimulates NF-κB and IRFs and the subsequent production of inflammatory mediators and type I IFN through a TRIF-dependent signaling cascade, which is required for eliminating viruses. However, there are reports indicating that TLR3 promotes pathogenesis rather than protecting. TLR3^–/–^ mice are more resistant to lethal WNV infection. Inflammation and neuropathology in TLR3^–/–^ mouse brains are also reduced (Wang et al., 2004). Similar results were observed in TLR3-deficient mice infected with IVA, which exhibited increased survival despite the higher viral loads in the lungs compared with the wild-type (Le Goffic et al., 2006). Based on the literature, the TLR3 signaling pathway plays an important role in the pathogenesis of HSE.

Poly(I:C) is a synthetic dsRNA, PAMP is associated with viral infection. Poly(I:C) is recognized by the antiviral pattern recognition receptors TLR3, RIG-I/MDA5 and PKR, thereby inducing signaling *via* multiple inflammatory pathways, including NF-kB and IRF. It has been reported that TLR3-deficient mice have a low response to poly(I:C) and a reduced production of inflammatory cytokines (Alexopoulou et al., 2001). Therefore, poly(I:C) served as a positive control in our study.

In the *in vitro* studies, the levels of mRNA and protein in the experimental group were significantly higher than those in the normal group, and the secretion of TNF-α, IL-6, and IFN-β was also increased. After the BV-2 cells were treated with corilagin, the inflammatory mediators were decreased to various degrees, while there were no significant changes in the ACV group compared with the model group. The results showed that corilagin inhibited the TLR3 signaling pathway. In order to confirm the efficacy of corilagin, lentiviral vectors or siRNAs were transfected into BV-2 cells to up-regulate or down-regulate, respectively, the expression of TLR3 and its downstream molecules. After stimulation with poly(I:C) or HSV-1, the TLR3 signaling pathway could be still inhibited by corilagin. In contrast, the levels of mRNA and proteins, and the secretion of TNF-α, IL-6, and IFN-β in the ACV group were slightly lower than those in the model group, yet not significantly. So in the *in vivo* experiments, the TLR3 signaling pathway was activated in mice with encephalitis. Compared with the ACV group, TLR3 and its downstream molecules in were significantly inhibited in the brain after treatment with the appropriate concentration of corilagin. The inflammatory lesions in brain tissues were also dramatically reduced, as assessed by histological analysis. Our research showed that corilagin inhibited TLR3 and its downstream molecules *in vivo* and that brain inflammatory damage was reduced. These findings show that corilagin has a distinct pharmacological mechanism from that of current drugs such as ACV.

In summary, our experiments *in vitro* and *in vivo* confirmed that corilagin can relieve the inflammatory response by inhibiting the TLR3 signaling pathway and reducing the release of inflammatory cytokines. Further researches on the mechanism of corilagin could be helpful for finding new ways to treat HSE.

## Ethics Statement

All animal research programs were adopted based on internationally recognized principles and Guidelines for the Care and use of the Laboratory Animals, Center of Huazhong University of Science and Technology. All experiments and animal care abided by internationally accepted principles and the Guidelines for the Care and Use of Laboratory Animals of Huazhong University of Science and Technology and were approved by the Ethics Committee of Union Hospital, Tongji Medical College, Huazhong University of Science and Technology.

## Author Contributions

LZ and Y-JG conceived the study. L-JL and S-JZ designed the experiments. S-JZ, PL, Y-QW, and Z-LC performed most of the experiments. Y-JW fed the animals and analyzed the mouse histopathology. J-BZ analyzed the data. S-JZ wrote the manuscript. All authors reviewed the manuscript.

## Conflict of Interest Statement

The authors declare that the research was conducted in the absence of any commercial or financial relationships that could be construed as a potential conflict of interest.
